# Self-Oscillation of Liquid Crystal Elastomer Fiber-Slide System Driven by Self-Flickering Light Source

**DOI:** 10.3390/polym16233298

**Published:** 2024-11-26

**Authors:** Dali Ge, Qingrui Hong, Xin Liu, Haiyi Liang

**Affiliations:** 1 School of Civil Engineering, Anhui Jianzhu University, Hefei 230601, China; 2 IAT-Chungu Joint Laboratory for Additive Manufacturing, Institute of Advanced Technology, University of Science and Technology of China, Hefei 241200, China; 3 CAS Key Laboratory of Mechanical Behavior and Design of Materials, Department of Modern Mechanics, University of Science and Technology of China, Hefei 230026, China

**Keywords:** self-oscillation, photo-responsive, liquid crystal elastomer, fiber, dynamic circuit

## Abstract

Self-oscillation, a control approach inspired by biological systems, demonstrates an autonomous, continuous, and regular response to constant external environmental stimuli. Until now, most self-oscillation systems have relied on a static external environment that continuously supplies energy, while materials typically absorb ambient energy only intermittently. In this article, we propose an innovative self-oscillation of liquid crystal elastomer (LCE) fiber-slide system driven by a self-flickering light source, which can efficiently regulate the energy input in sync with the self-oscillating behavior under constant voltage. This system primarily consists of a photo-responsive LCE fiber, a slider that includes a conductive segment and an insulating segment, a light source, and a conductive track. Using the dynamic LCE model, we derive the governing equation for the motion of the LCE fiber-slider system. Numerical simulations show that the LCE fiber-slide system under constant voltage exhibits two distinct motion phases, namely the stationary phase and the self-oscillation phase. The self-oscillation occurs due to the photo-induced contraction of the LCE fiber when the light source is activated. We also investigate the critical conditions required to initiate self-oscillation, and examine key system parameters influencing its frequency and amplitude. Unlike the continuous energy release from the static environmental field in most self-oscillation systems, our LCE fiber-slide self-oscillation system is driven by a self-flickering light source, which dynamically adjusts the energy input under a constant voltage to synchronize with the self-oscillating behavior. Our design features advantages such as spontaneous periodic lighting, a simple structure, energy efficiency, and ease of operation. It shows significant promise for dynamic circuit systems, monitoring devices, and optical applications.

## 1. Introduction

A diverse range of artificial materials has been meticulously engineered to respond effectively to various stimuli, including light [1,2], heat [3,4], chemical substances [5,6], magnetic fields [7], electric fields [8,9], humidity [10,11], and more. Many artificial materials, including liquid crystal elastomers (LCEs) [1,2], polyvinylidene fluoride (PVDF) [3], hydrogels [4,6], and carbon nitride polymers [11], possess a remarkable ability to undergo reversible shape changes when subjected to appropriate stimuli, enabling them to adapt dynamically to their environments [12,13]. In some cases, they can even exhibit controlled movement, which allows for complex interactions with surrounding conditions [14]. This unique feature not only enhances their functionality but also paves the way for their application in the development of intelligent and autonomous devices, including wearable health monitors, autonomous delivery robots, smart home assistants, and energy harvesters.

However, a significant challenge in the application of autonomous devices is that, in numerous instances, the associated external factors demand proper control strategies to achieve consistent and predictable dynamic behavior [15,16,17]. Conventional control strategies include electronic control, sensor-based control, external device control, and even manual control [18,19]. These strategies typically depend on external devices to send control signals, sensors to measure changes in physical quantities and provide real-time feedback, or manual switches to adjust the system. Nonetheless, conventional control strategies often have limitations, such as reliance on human intervention or the need for complex control systems. Additionally, they generally lack intelligent functionality. Given the diverse and complex real application scenarios, conventional control strategies for designing or utilizing autonomous devices may face numerous challenges.

The intelligent and autonomous features of biological organisms serve as a rich source of inspiration for designing autonomous devices. Self-oscillation in these organisms—exemplified by the involuntary beating of the heart, neural impulses, and cell cycling—demonstrates an autonomous, continuous, and regular response to constant external environmental stimuli, without the need for additional on/off switching signals [20,21]. The amplitude and frequency of their oscillations are determined by their intrinsic characteristics, exhibiting remarkable robustness [22,23]. Given these advantages, self-oscillation systems show great promise for use in autonomous robotics [24,25,26], energy-absorbing devices [27,28], mass transport systems [29,30], mechano-logistic devices [31], optical devices [32,33], and more.

To satisfy the multi-functional needs of different applications, a vast number of self-sustained motion modes have been constructed, such as bending [34,35,36,37,38], buckling [6,39,40,41,42], rocking [43,44,45], jumping [46,47], rolling [24,48,49,50,51,52,53,54], rotation [23,54,55,56,57,58,59], spinning [60,61], swimming [62,63], swinging [64,65], stretching and shrinking [34,66,67], snap-through [68,69], torsion [23,29,34,70], vibration [34,71,72,73,74], and even the harmonized motion of several interconnected self-oscillating systems [75,76]. Such self-sustained movements are often the result of nonlinear feedback processes, including phenomena like self-shadowing [40,44], the interaction between large deformations and chemical reactions [6], photothermal evaporation processes [37], and gradients in surface tension induced by photothermal effects [77]. 

Along with many other similar artificial materials, LCE is a type of responsive material that combines the elastic properties of long polymer chains with the ordered structure of liquid crystal (LC) mesogens. Due to the ability of these mesogens to reversibly switch between isotropic and anisotropic states, LCE can respond to various physical stimuli, including magnetic fields, heat, electricity, and light, resulting in significant macroscopic deformations [18,78]. Among these stimuli, optical excitation stands out due to its distinct benefits, including the ability to drive systems without physical contact, flexibility in programming, ease of control, high precision, and environmental sustainability [16,21,28]. When subjected to optical stimuli, optically responsive LCEs demonstrate rapid response times, substantial intrinsic deformation, and reversible shape changes. These distinctive characteristics facilitate self-oscillation in diverse forms, leading to considerable advancements in the development of LCE-based optically powered self-oscillation systems [24,29,32,33,40,41,42].

So far, most self-oscillation systems have been developed based on the continuous energy release from a static environmental field [40,41,42,52,56,62]. However, in these systems, materials tend to absorb ambient energy only intermittently, leading to considerable energy waste. To tackle this issue, we propose a novel self-oscillation of the LCE fiber-slide system driven by a self-flickering light source, which effectively regulates the energy source in harmony with the self-oscillating behavior. This system primarily consists of a photo-responsive LCE fiber, a slider that includes a conductive segment and an insulating segment, a light source, a conductive track, and direct current (DC) power supply. In contrast to the continuous energy release from the static environmental field in most self-oscillation systems, our system features a dynamic, self-flickering light source that interacts with the oscillation of the slider when exposed to a constant voltage, enabling periodic switching over time. This setup offers advantages such as spontaneous periodic lighting, a simple structure, energy efficiency, and ease of operation. It holds promise for constructing dynamic circuit systems, monitoring and warning devices, and optical devices, as well as other advanced technologies.

The paper is organized in the following way. In Section 2, a self-oscillating LCE fiber-slide system driven by a self-flickering light source is introduced, along with the formulation of its dynamic governing equations. In Section 3, numerical simulations are employed to explore two phases: the stationary phase and the self-oscillation phase, with a detailed revelation of the mechanisms underlying self-oscillation. In Section 4, the impact of system parameters on the self-oscillation behavior is extensively studied. Finally, conclusions are drawn in Section 5.

## 2. Theoretical Framework

In this section, we present an analysis of the self-oscillation in an LCE fiber-slide system powered by a self-flickering light source. Key topics include the theoretical framework for the self-oscillating LCE fiber-slider system, the dynamics of the cis molecule concentration evolution within the LCE fiber, the process of nondimensionalization, and the approach for solving the differential governing equations that feature variable coefficients.

### 2.1. Self-Oscillating Dynamic of the LCE Fiber-Slide System

**Figure 1:**
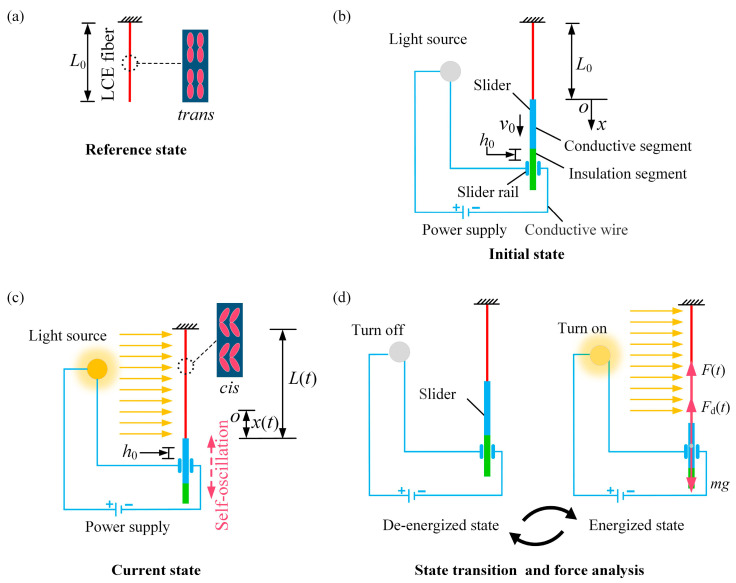
Schematic diagram of a self-oscillation of LCE fiber-slide system driven by self-flickering light source. (**a**) Reference state. (**b**) Initial state. (**c**) Current state. (**d**) State transition and force analysis.

Figure 1 illustrates a self-oscillation of the LCE fiber-slide system driven by a self-flickering light source. The system consists of a photo-responsive LCE fiber with an initial length of $L_{0}$ in a stress-free state, a long slider with a mass m that consists of a conductive segment and an insulating segment, a DC power supply, a light source, conductive wires, and a conductive track. In the reference state, the azobenzene molecules in the LCE fiber are aligned along the length of the fiber, maintaining a straight trans state, as illustrated in Figure 1a. It is widely recognized that exposure to light prompts the azobenzene molecules to shift from the straight trans state to a bent cis state, leading to reversible photo-induced contraction along the fiber’s length. This contraction can subsequently return to its original state in the absence of light.

One terminus of the LCE fiber is anchored at a suspension point, with its opposite extremity connected to a slider. The conductive segment of the slider is represented by the blue zone, and the insulating segment is highlighted in the green zone. The slider is situated within the conductive track. In a stress-neutral condition, the upper end of the insulating segment of the slider is positioned $h_{0}$ above the conductive track, as shown in Figure 1b. Conductive wires connect the power supply, light source, and both ends of the conductive track in series. Together, the power supply, light source, conducting wires, slider, and conductive track form a complete circuit system. The state of the circuit system, whether energized or de-energized, is determined by whether the conductive segment of the slider is in contact with the track or if the insulated segment of the slider is in contact with the track, as depicted in Figure 1d. In the starting configuration, the slider is set free under specific initial conditions. Influenced by gravity, the slider begins to move downward. To accurately describe the instantaneous displacement x(t) of the slider at time t, we establish a coordinate system with the reference O positioned at the bottom terminus of the fiber in its stress-free state, as illustrated in Figure 1b. At this moment, the LCE fiber is stretched to a length L(t) and experiences a tensile force F(t). As the slider’s displacement increases and surpasses $h_{0}$, its conductive segment comes into contact with the conductive track, energizing the circuit and activating the light source, which exposes the entire length of the LCE fiber to light, as shown in Figure 1c. Consequently, the LCE fiber contracts along its length, resulting in a reduction in its overall length. It is essential to understand that the tensile force F(t), arising from the combined effects of the fiber’s stretched state and the photo-induced contraction, causes the slider to decelerate and retract under the combined action of the damping force $F_{d}(t)$. As the slider moves upward and its insulating segment contacts the conductive track, the circuit enters a de-energized state, turning off the light source and allowing the LCE fiber to return to its original state in the dark. Concurrently, the decrease in tensile force F(t) results in the slider accelerating downward, eventually causing it to move downward again. Thus, the slider may oscillate continuously over time, accompanied by the dynamic flickering of the light source.

During the motion of the slider, we assume that the frictional force generated between the slider and the track can be negligible. Under this assumption, the slider is subjected to the tensile force F(t) of the LCE fiber, gravity mg, and the damping force $F_{d}(t)$, as illustrated in Figure 1d. Then, the equation governing the motion of the slider can be derived as follows:

(1) $$m\ddot{x}=mg-F\left(t\right)-F_{d}\left(t\right),$$

with the initial conditions

(2) $$x\left(t\right)=x_{0}\;\mathrm{and}\;\dot{x}=v_{0}\mathrm{at}\;t=0,$$

where g is the gravity acceleration, $\ddot{x}$ represents the acceleration of the slider, and $F_{d}(t)$ represents the damping force. It is essential to understand that, in practical situations, the damping force $F_{d}(t)$ is intricately related to the velocity of the system. To facilitate subsequent calculations, the damping force $F_{d}(t)$ is assumed to be directly proportional to the velocity $\dot{x}$ of the slider, with the force acting in the reverse direction to the motion, which can be given as following equation:

(3) $$F_{d}\left(t\right)=\beta \dot{x},$$

where  β is the damping coefficient.

To determine the tensile force F(t) in Equation (1), an assumption being made is that the tensile force is uniformly distributed and varies in direct proportional to the elastic strain $\varepsilon_{e}\left(t\right)$, i.e.,

(4) $$F\left(t\right)=kL_{0}\varepsilon_{e}\left(t\right),$$

where k is the stiffness coefficient of the LCE fiber. Notably, the elastic strain $\varepsilon_{e}\left(t\right)$ in the LCE fiber is assumed to be homogeneous and can be represented as a linear combination of the overall strain $\varepsilon_{tot}\left(t\right)$ and the photo-induced contraction $\varepsilon \left(t\right)$. Considering the geometric relationship between the overall deformation of the LCE fiber and the displacement of the slider, the overall strain can be written as the following:

(5) $$\varepsilon_{tot}\left(t\right)=x\left(t\right)/L_{0}.$$

Consequently, the tensile force can be given as the following:

(6) $$F\left(t\right)=k\left[x\left(t\right)-\varepsilon \left(t\right)L_{0}\right].$$

To determine the tensile force in Equation (6), it is essential to first estimate the photo-induced contraction $\varepsilon \left(t\right)$, the details of which are provided in the next section.

### 2.2. Dynamic LCE Model

The photo-induced contraction strain $\varepsilon \left(t\right)$ is closely related to the concentration $\varphi \left(t\right)$ of cis molecules within the LCE fiber. To simplify, we assume that the concentration is significantly smaller than 1. Consequently, by applying a Taylor expansion and considering only the first-order term, we can express the photo-induced contraction strain $\varepsilon \left(t\right)$ as being proportional to the cis molecule’s concentration, i.e.,

(7) $$\varepsilon \left(t\right)=-C_{0}\varphi \left(t\right),$$

where $C_{0}$ represents the contraction coefficient. 

According to the previous work of Yu et al., trans-to-cis molecules in LCE can be induced by UV or laser light with wavelengths shorter than 400 nm [78]. Furthermore, the concentration $\varphi \left(t\right)$ of the cis molecules is influenced by thermal excitation from trans molecules to cis molecules, and the light-driven relaxation from cis to trans [20,79,80]. Typically, the thermal excitation from trans molecules to cis molecules is regarded as insignificant in comparison to the photo-induced processes [78,81]. To determine the concentration $\varphi \left(t\right)$ of cis molecules within the LCE fiber, we employ the well-established dynamic LCE model proposed by Finkelmann et al. [20,79,80]. The concentration $\varphi \left(t\right)$ is governed by the following equation:

(8) $$\frac{d\varphi \left(t\right)}{dt}=\eta_{0}I(t)\left[1-\varphi \left(t\right)\right]-\tau^{-1}\varphi \left(t\right),$$

where τ is the thermal relaxation time from cis molecules to trans molecules, $\eta_{0}$ is the light absorption constant, and the light intensity I(t) is determined by the current displacement of the slider. When the LCE fiber is illuminated due to the circuit being turned on (i.e., $x\left(t\right)\;$>$\;h_{0}$), the light intensity is set at I(t) = $I_{0}$. Clearly, when the circuit is turned off (i.e., $x\left(t\right)\;$≤ $h_{0}$), the light intensity can be set to I(t) = 0.

### 2.3. Nondimensionalization

To aid in exploring the dynamic characteristics of the self-oscillation of the LCE fiber-slider system, the following dimensionless quantities are defined: ${\bar{I}}_{0}=\eta_{0}I_{0}\tau_{0}$, $\bar{I}=\eta_{0}I\tau_{0}$, $\bar{t}=t/\tau$, $\bar{x}=x/L_{0}$, ${\bar{x}}_{0}=x_{0}/L_{0}$, ${\bar{v}}_{0}=\tau v_{0}/L_{0}$, $\bar{\dot{x}}=\dot{x}\tau /L_{0}$, $\bar{\ddot{x}}=\ddot{x}\tau^{2}/L_{0}$, $\bar{F}=F\tau^{2}/mL_{0}$, ${\bar{F}}_{d}=F_{d}\tau^{2}/mL_{0}$, $\bar{g}=g\tau^{2}/L_{0},$ $\bar{k}=k\tau^{2}/m$, $\bar{\beta }=\beta \tau /m$*,* and ${\bar{h}}_{0}=h/L_{0}$. The governing Equations (1)–(6) for the slider can be rewritten in the following form:

(9) $$\bar{\ddot{x}}=\bar{g}-\bar{F}\left(\bar{t}\right)-{\bar{F}}_{d}\left(\bar{t}\right),$$

with the initial conditions

(10) $$\bar{x}\left(\bar{t}\right)={\bar{x}}_{0}\mathrm{and}\;\bar{\dot{x}}={\bar{v}}_{0}\;\mathrm{at}\;\bar{t}=0.$$

The dimensionless damping force can be calculated as

(11) $${\bar{F}}_{d}\left(\bar{t}\right)=\bar{\beta }\bar{\dot{x}}.$$

The tensile force of the LCE fiber can be determined as follows:

(12) $$\bar{F}\left(\bar{t}\right)=\bar{k}\left[\bar{x}\left(\bar{t}\right)+C_{0}\varphi \left(\bar{t}\right)\right].$$

Similarly, the concentration can be determined by

(13) $$\frac{d\varphi \left(\bar{t}\right)}{d\bar{t}}=\bar{I}(\bar{t})\left[1-\varphi \left(\bar{t}\right)\right]-\varphi \left(\bar{t}\right),$$

where $\bar{I}(\bar{t})$ = ${\bar{I}}_{0}$ for $\bar{x}\left(\bar{t}\right)$ > ${\bar{h}}_{0}$; otherwise, $\bar{I}(\bar{t})$ = 0.

Equations (7) and (11) govern the self-oscillating behavior of the LCE fiber-slider system. To tackle these complex differential equations with variable coefficients, we employ the Runge–Kutta method for numerical calculations in MATLAB 2023b software. At time ${\bar{t}}_{i}$, the concentration $\varphi \left({\bar{t}}_{i}\right)$ of the *cis* molecules and the displacement $\bar{x}\left({\bar{t}}_{i}\right)$ can be used to determine the current tensile force $\bar{F}\left({\bar{t}}_{i}\right)$ in the LCE fiber according to Equation (12). Subsequently, the displacement $\bar{x}\left({\bar{t}}_{i+1}\right)$ of the slider at time ${\bar{t}}_{i+1}$ can be determined using Equation (9), while the light intensity $\bar{I}({\bar{t}}_{i+1})$ is simultaneously estimated. Subsequently, the concentration $\varphi \left({\bar{t}}_{i+1}\right)\;$ of the *cis* molecules is derived from Equation (13). In summary, by iterating this process, the self-oscillation behavior of the LCE fiber-slider system can be numerically obtained for specified parameters: ${\bar{I}}_{0}$, $C_{0}$, $\bar{k}$, ${\bar{x}}_{0}$, ${\bar{v}}_{0}$, ${\bar{h}}_{0}$, $\bar{\beta }$, and $\bar{g}$. It is worth mentioning that ${\bar{x}}_{0}$ can be converted into the corresponding ${\bar{v}}_{0}$ by utilizing the system’s energy relationship. To be concise, we fix ${\bar{x}}_{0}=0$ in the calculation.

## 3. Two Motion Phases and Mechanism of Self-Oscillation

In this section, we begin by discussing two distinct motion phases of the self-oscillation of the LCE fiber-slider system driven by a self-flickering light source, specifically the stationary phase and the self-oscillation phase. Following this, we offer a comprehensive explanation of the mechanism underlying the self-oscillation.

### 3.1. Two Motion Phases

To investigate the self-oscillation behavior of the self-oscillation of the LCE fiber-slider system, we should first establish the specific values of the dimensionless parameters in the model. Typical material properties and geometric parameters are provided in Table 1 [81,82,83]. The corresponding dimensionless parameters are also detailed in Table 2. These parameter values are utilized in the subsequent analysis of the self-oscillation behavior of the LCE fiber-slider system.

**Figure 2:**
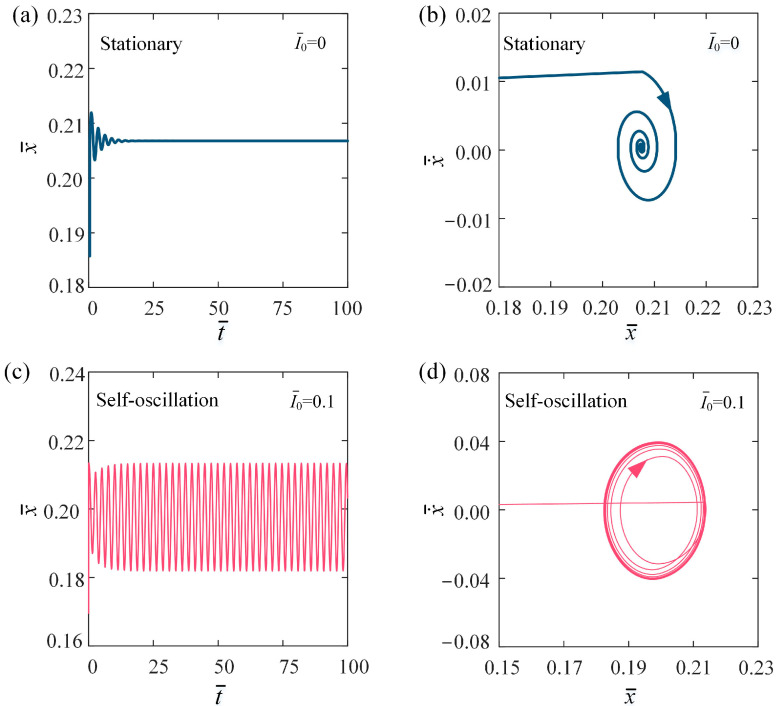
Temporal behavior of displacement and phase trajectory curves for two motion phases. (**a**) Displacement temporal behavior with ${\bar{I}}_{0}$ = 0; (**b**) phase trajectory with ${\bar{I}}_{0}$ = 0; (**c**) displacement temporal behavior with ${\bar{I}}_{0}$ = 0.1; and (**d**) phase trajectory with ${\bar{I}}_{0}$ = 0.1. Other parameters are $C_{0}$ = 0.2, $\bar{\beta }$ = 0.1, $\bar{k}$ = 5.8, ${\bar{v}}_{0}$ = 0.1, $\bar{g}$ = 1.2, and ${\bar{h}}_{0}$ = 0.2. Under constant voltage, the LCE fiber-slide system driven by self-flickering light source exhibits two distinct motion phases: the stationary phase and the self-oscillation phase.

Figure 2 illustrates two distinct motion phases of the self-oscillation in the LCE fiber-slider system: the stationary phase for ${\bar{I}}_{0}$ = 0, as shown in Figure 2a,b, and the self-oscillation phase for ${\bar{I}}_{0}$ ≠ 0, as shown in Figure 2c,d. The other parameters are set as follows: $C_{0}$ = 0.2, $\bar{\beta }$ = 0.1, $\bar{k}$ = 5.8, ${\bar{v}}_{0}$ = 0.1, $\bar{g}$ = 1.2, and ${\bar{h}}_{0}$ = 0.2. Through the numerical solution of Equations (7) and (11), we can derive both the temporal behavior of displacement and phase trajectory curves of the slider. In the scenario where ${\bar{I}}_{0}$ = 0, the slider experiences vertical oscillations driven by gravitational forces and the tension within the LCE fiber. However, due to damping effects, the amplitude and velocity of the oscillation gradually diminish, causing the slider to ultimately settle into a stationary equilibrium position, which we term the stationary phase, as illustrated Figure 2a,b. Conversely, when ${\bar{I}}_{0}$ = 0.1, the slider continues to oscillate due to the effects of gravity and the tensile force. During the alternating contact between the conductive and insulating segments of the slider and the conductive track, the dynamic circuit causes the light source to switch between the activated and deactivated states, resulting in self-flickering. The incorporation of a dynamic electrical circuit enables the LCE material to convert light energy from the self-flickering light source, effectively offsetting energy losses due to dissipation. As a result, the amplitude of the slider’s vibrations increases over time until it stabilizes. With a constant voltage supplied by the DC power source, the slider eventually undergoes continuous periodic oscillations, entering what is known as the self-oscillation phase, as shown in Figure 2c,d.

### 3.2. Mechanisms of Self-Oscillation

**Figure 3:**
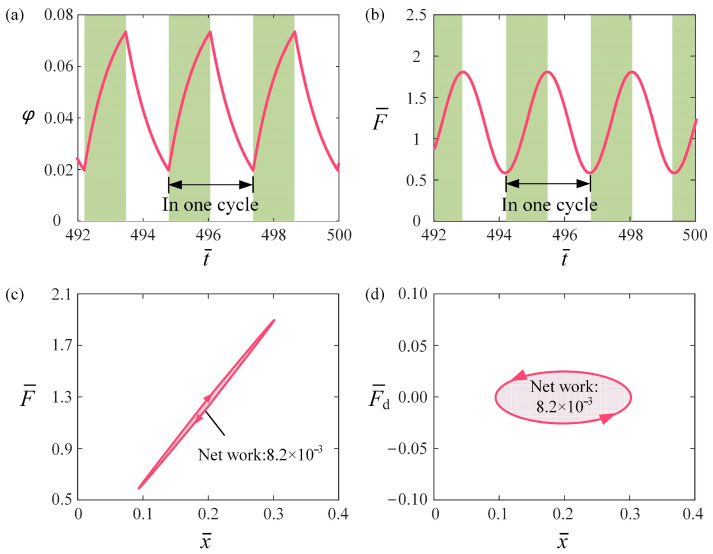
Mechanism of the self-oscillation of the LCE fiber-slider system driven by self-flickering light source for the typical case in Figure 2c,d. (**a**) Variation of concentration of *cis* molecules over $\bar{t}$; (**b**) Variation of LCE fiber $\bar{F}$ over $\bar{t}$. (**c**) The relationship between $\bar{F}$ and $\bar{x}$, and (**d**) relationship between ${\bar{F}}_{d}$ and $\bar{x}$. The damping dissipation is balanced by the energy input from the tensile force, thereby maintaining stable self-oscillation.

To elucidate the self-oscillation mechanism of the self-oscillation of the LCE fiber-slider system, Figure 3 presents several key physical quantities of the fiber-slider system for $C_{0}$ = 0.2, ${\bar{I}}_{0}=0.1$, $\bar{\beta }$ = 0.1, $\bar{k}$ = 5.8, ${\bar{v}}_{0}$ = 0.1, $\bar{g}$ = 1.2, and ${\bar{h}}_{0}$ = 0.2. The green-highlighted regions in Figure 3a,b signify that the circuit is energized and the LCE fiber is subjected to light. As the slider moves downward from its initial state, the length of the LCE fiber increases, and then the slider’s conductive segment makes contact with the conductive track, causing the circuit to be in an energized state. At this point, φ of the LCE fiber gradually increases over time, leading to a decrease in the length of the fiber and causing the slider to move upward. Once the insulating segment of the slider makes contact with the conductive track, the circuit enters a de-energized state. At this stage, φ gradually decreases over time, resulting in an elongation of the LCE fiber and causing the slider to move downward again. As a result, the concentration of the *cis* molecules exhibits a periodic change over time during the slider’s motion, as shown in Figure 3a. Moreover, it is clear from Equation (4) that the tensile force $\bar{F}$ also exhibits a periodic variation over time, as depicted Figure 3b. This ultimately results in the displacement of the slider, which similarly demonstrates periodic changes.

Figure 3c describes the variation of the tensile force with displacement. The curve representing the tensile force $\bar{F}$ against displacement $\bar{x}$ forms a closed loop in a clockwise direction. The area enclosed by this loop signifies the net work performed by tensile force on the system, representing the energy input, which is calculated to be 0.0082 over one cycle. Figure 3d depicts the variation of the damping force with displacement, forming a closed loop in a counterclockwise direction. The area within this loop corresponds to the damping dissipation, also calculated to be 0.0082—exactly matching the net work done by the tensile force. As a result, during the self-oscillation of the LCE fiber-slider system driven by the flickering light source, the damping dissipation is balanced by the energy input from the tensile force, thereby maintaining the stable self-oscillation of the system.

## 4. Parameter Analysis

In the preceding analysis of the self-oscillation in an LCE fiber-slide system powered by a self-flickering light source, seven dimensionless system parameters were identified: ${\bar{I}}_{0}$, $C_{0}$, $\bar{\beta }$, $\bar{k}$, ${\bar{v}}_{0}$, $\bar{g}$, and ${\bar{h}}_{0}$. This section aims to investigate how different system parameters influence the triggering conditions for self-oscillation, as well as their effects on the dimensionless amplitude A and frequency f of self-oscillation.

### 4.1. Impact of the Light Intensity

**Figure 4:**
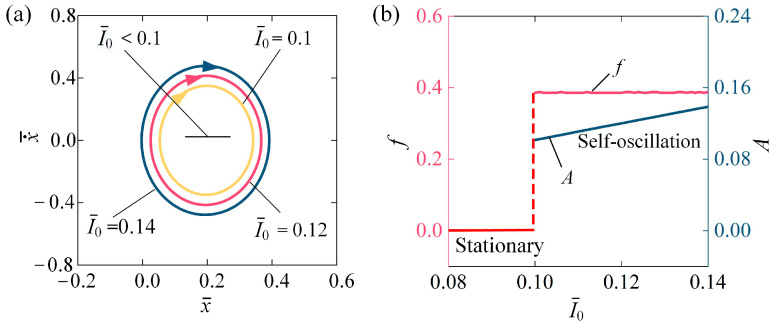
Influence of light intensity on self-oscillation of LCE fiber-slide system, for $C_{0}$ = 0.2, $\bar{\beta }$ = 0.1, $\bar{k}$ = 5.8, ${\bar{v}}_{0}$ = 0.1, $\bar{g}$ = 1.2, and ${\bar{h}}_{0}$ = 0.2. (**a**) Stable cycles. (**b**) f and A. As ${\bar{I}}_{0}$ increases, the A tends to rise, while the f remains relatively stable.

Figure 4 illustrates the impact of light intensity ${\bar{I}}_{0}$ on the self-oscillation behavior of the LCE fiber-slide system. We set $C_{0}$ = 0.2, $\bar{\beta }$ = 0.1, $\bar{k}$ = 5.8, ${\bar{v}}_{0}$ = 0.1, $\bar{g}$ = 1.2, and ${\bar{h}}_{0}$ = 0.2. Figure 4a depicts the stable cycles for various ${\bar{I}}_{0}$. The threshold value for initiating self-oscillation in the system is approximately ${\bar{I}}_{0}$ = 0.1. Specifically, when $\;{\bar{I}}_{0}$ < 0.1, the system stays in a stationary phase, whereas at ${\bar{I}}_{0}$ = 0.1, ${\bar{I}}_{0}$ = 0.12, and ${\bar{I}}_{0}$ = 0.14, self-oscillation occurs. Figure 4b presents the relationship between the frequency f and amplitude A of self-oscillation and light intensity ${\bar{I}}_{0}$. As ${\bar{I}}_{0}$ increases, A tends to rise, while f remains relatively stable. As ${\bar{I}}_{0}$ increases, more energy is absorbed and converted into mechanical energy, leading to a rise in A. On the other hand, the stability in frequency can be attributed to the fact that the oscillation frequency of the LCE fiber-slide system is mainly governed by its inherent mechanical characteristics. Thus, increasing the ${\bar{I}}_{0}$ can enhance the practical use of self-oscillation in the LCE fiber-slide system.

### 4.2. Impact of the Contraction Coefficient

**Figure 5:**
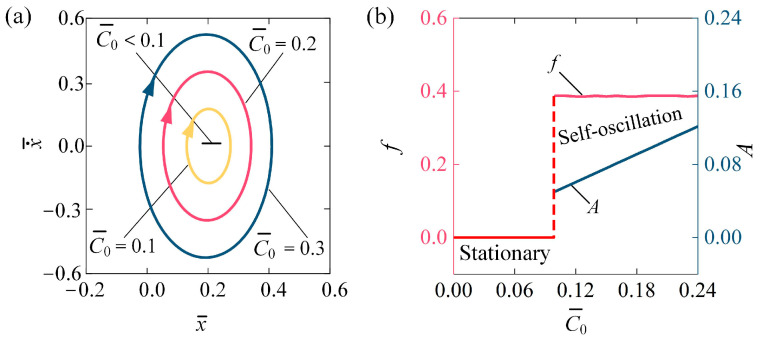
Influence of contraction coefficient on self-oscillation of LCE fiber-slide system, for ${\bar{I}}_{0}$ = 0.1, $\bar{\beta }$ = 0.1, $\bar{k}$ = 5.8, ${\bar{v}}_{0}$ = 0.1, $\bar{g}$ = 1.2, and ${\bar{h}}_{0}$ = 0.2. (**a**) Stable cycles. (**b**) f and A. As the contraction coefficient increases, the  A shows a distinct increase, while the f stays largely unaffected.

Figure 5 illustrates how the contraction coefficient $C_{0}$ impacts the self-oscillating behavior of the LCE fiber-slide system for ${\bar{I}}_{0}$ = 0.1, $\bar{\beta }$ = 0.1, $\bar{k}$ = 5.8, ${\bar{v}}_{0}$ = 0.1, $\bar{g}$ = 1.2, and ${\bar{h}}_{0}$ = 0.2. Figure 5a shows the stable cycles for different $C_{0}$. The critical $C_{0}$ to trigger the self-oscillation of the LCE fiber-slide system is approximately $C_{0}$ = 0.1. That is, for $C_{0}$ < 0.1, the system is in a stationary phase, while for $C_{0}$ = 0.1, $C_{0}$ = 0.2, and $C_{0}$ = 0.3, the system exhibits a self-oscillation phase. Figure 5b demonstrates how the f and A vary with $C_{0}$. As $C_{0}$ rises, there is a noticeable increase in A, while the f remains relatively unchanged. Equation (5) supports the observation that as $C_{0}$ increases, the photo-induced contraction also increases, resulting in an increase in tensile force; consequently, the amplitude of self-oscillation increases. The findings indicate that boosting the contraction coefficient of the LCE materials can enhance the efficiency of converting light energy into mechanical energy.

### 4.3. Impact of the Damping Coefficient

**Figure 6:**
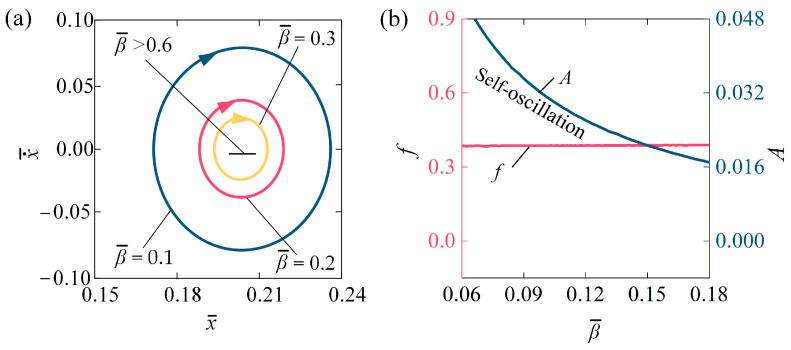
Influence of damping coefficient on self-oscillation of LCE fiber-slide system, for ${\bar{I}}_{0}$ = 0.1, $C_{0}$ = 0.2, $\bar{k}$ = 5.8, ${\bar{v}}_{0}$ = 0.1, $\bar{g}$ = 1.2, and ${\bar{h}}_{0}$ = 0.2. (**a**) Stable cycles. (**b**) f and A. With the increase in damping coefficient, the A presents a decreasing trend, while the f remains nearly constant.

Figure 6 describes the influence of damping coefficient $\bar{\beta }$ on the self-oscillating behavior of the LCE fiber-slide system. In the analysis, we set ${\bar{I}}_{0}$ = 0.1, $C_{0}$ = 0.2, $\bar{k}$ = 5.8, ${\bar{v}}_{0}$ = 0.1, $\bar{g}$ = 1.2, and ${\bar{h}}_{0}$ = 0.2. Figure 6a illustrates the stable cycles associated with various damping coefficients. The threshold value for initiating self-oscillation is approximately $\bar{\beta }$ = 0.6. In other terms, for $\bar{\beta }$ > 0.6, the system is in a stationary phase, while for $\bar{\beta }$ = 0.1, $\bar{\beta }$ = 0.2, and $\bar{\beta }$ = 0.3, the system is in a self-oscillation phase. Figure 6b shows how the f  and A change with respect to $\bar{\beta }$. As $\bar{\beta }$ increases, A presents a decreasing trend, while f  remains almost unchanged. When $\bar{\beta }$ surpasses 0.6, the energy input becomes inadequate to counterbalance the energy lost due to damping, resulting in the system reaching a stationary phase. Consequently, to enhance energy harvesting in engineering applications, a strategic reduction in the damping coefficient is an effective approach to improve light energy absorption during the self-oscillation of the LCE fiber-slide system.

### 4.4. Impact of the Stiffness Coefficient

**Figure 7:**
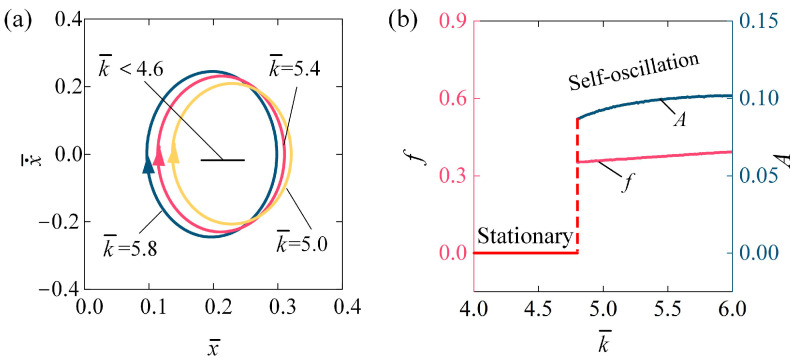
Influence of stiffness coefficient on self-oscillation of LCE fiber-slide system, for ${\bar{I}}_{0}$ = 0.1, $C_{0}$ = 0.2, $\bar{\beta }$ = 0.1, ${\bar{v}}_{0}$ = 0.1, $\bar{g}$ = 1.2, and ${\bar{h}}_{0}$ = 0.2. (**a**) Stable cycles. (**b**) f and A. As the stiffness coefficient increases, both the a f and A exhibit an upward trend.

Figure 7 demonstrates the influence of the stiffness coefficient $\bar{k}$ on the self-oscillating behavior of the LCE fiber-slide system, with parameters set to ${\bar{I}}_{0}$ = 0.1, $C_{0}$ = 0.2, $\bar{\beta }$ = 0.1, ${\bar{v}}_{0}$ = 0.1, $\bar{g}$ = 1.2, and ${\bar{h}}_{0}$ = 0.2. The stable cycles for various stiffness coefficients are depicted in Figure 7a. The critical threshold for initiating self-oscillation is approximately $\bar{k}$ = 4.6. In this context, when $\bar{k}$ < 4.6, the system remains static; however, at $\bar{k}$ = 5.0, $\bar{k}$ = 5.4, and $\bar{k}$ = 5.8, the system exhibits self-oscillation. For lower values of $\bar{k}$, the tensile force is minimal, leading to insufficient net work done on the system. Consequently, the energy supplied is insufficient to counteract the damping losses required to maintain self-oscillation. Figure 7b illustrates the relationship between frequency, amplitude of self-oscillation, and the stiffness coefficient $\bar{k}$. As $\bar{k}$ increases, both the A and f exhibit an increasing pattern. Based on the derivation above, the tensile force of the LCE fiber rises with an increase in $\bar{k}$. As a result, the net work performed by this tensile force during each cycle also increases. A higher stiffness coefficient in the LCE fiber improves its ability to effectively convert light energy into mechanical energy within the self-oscillating system. Consequently, increasing the stiffness coefficient of the LCE material can enhance both the frequency and amplitude, making it better suited for a range of engineering applications.

### 4.5. Impact of the Initial Condition

**Figure 8:**
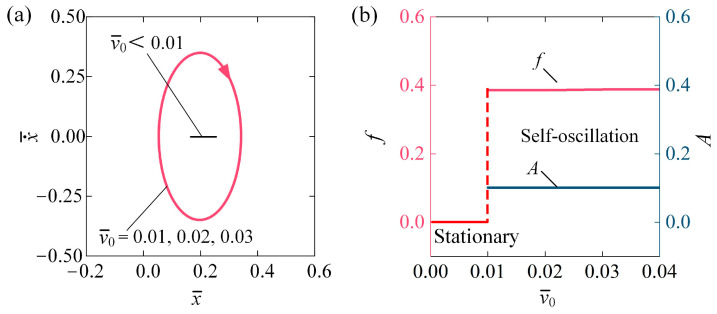
Influence of initial velocity on self-oscillation of LCE fiber-slide system, for ${\bar{I}}_{0}$ = 0.1, $C_{0}$ = 0.2, $\bar{\beta }$ = 0.1, $\bar{k}$ = 5.8, $\bar{g}$ = 1.2, and ${\bar{h}}_{0}$ = 0.2. (**a**) Stable cycles. (**b**) f and A. Initial conditions do not influence the A and f of self-oscillation.

Figure 8 illustrates the effect of initial velocity ${\bar{v}}_{0}$ on the self-oscillation of the LCE fiber-slide system for ${\bar{I}}_{0}$ = 0.1, $C_{0}$ = 0.2, $\bar{\beta }$ = 0.1, $\bar{k}$ = 5.8, $\bar{g}$ = 1.2, and ${\bar{h}}_{0}$ = 0.2. The stable cycles for different initial velocities are presented in Figure 8a. A critical velocity ${\bar{v}}_{0}$ of approximately 0.01 marks the transition between the stationary phase and the self-oscillating phase. For ${\bar{v}}_{0}$ < 0.01, the system stays in the stationary phase because the energy input is insufficient to counterbalance the energy lost to damping. The self-oscillating phase is activated at ${\bar{v}}_{0}$ = 0.01, ${\bar{v}}_{0}$ = 0.02, and ${\bar{v}}_{0}$ = 0.03, each resulting in the same stable cycles, as shown in Figure 8a. Figure 8b displays the f and A of self-oscillation in relation to ${\bar{v}}_{0}$, respectively. Notably, both f and A remain unchanged with varying ${\bar{v}}_{0}$. Given that the effects of initial displacement ${\bar{x}}_{0}$ and initial velocity ${\bar{v}}_{0}$ can be equivalently transformed through energy conversion, we can conclude that the initial conditions only influence whether self-excited oscillation is triggered; however, they do not affect the amplitude and frequency of self-oscillation, which are intrinsic properties of the system [22].

### 4.6. Impact of the Gravitational Acceleration

**Figure 9:**
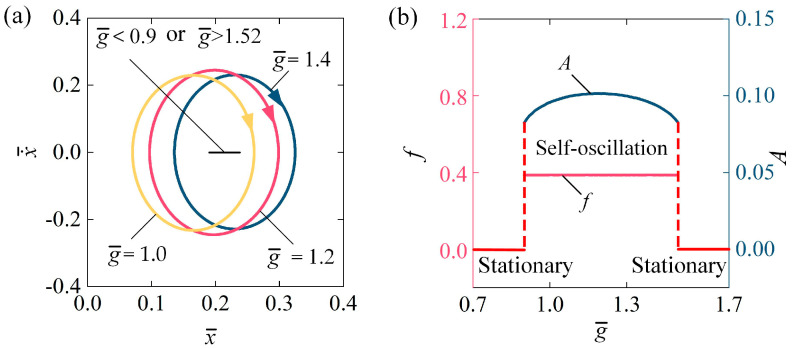
Influence of gravitational acceleration on self-oscillation of LCE fiber-slide system, for ${\bar{I}}_{0}$ = 0.1, $C_{0}$ = 0.2, $\bar{\beta }$ = 0.1, ${\bar{v}}_{0}$ = 0.1, $\bar{k}$ = 5.8, and ${\bar{h}}_{0}$ = 0.2. (**a**) Stable cycles. (**b**) f and A. As $\bar{g}$ increases, the A initially rises and then falls, while f has barely changed at all.

Figure 9 describes the influence of gravitational acceleration $\bar{g}$ on the self-oscillation of the LCE fiber-slide system. In the calculation, we set ${\bar{I}}_{0}$ = 0.1, $C_{0}$ = 0.2, $\bar{\beta }$ = 0.1, ${\bar{v}}_{0}$ = 0.1, $\bar{k}$ = 5.8, and ${\bar{h}}_{0}$ = 0.2. The stable cycles for various $\bar{g}$ are depicted Figure 9a. The range of gravitational acceleration required to initiate self-oscillation in the LCE fiber-slide system is approximately $0.9\leq \bar{g}\leq 1.52$. In other terms, for $\bar{g}$ < 0.9 or $\bar{g}$ > 1.52, the system is in a stationary phase. For $\bar{g}$ = 1.0, $\bar{g}$ = 1.2, and $\bar{g}$ = 1.4, the system exhibits a self-oscillation state. This outcome can be attributed to the balance between the energy input and the energy lost due to damping. Figure 9b illustrates how the f and A of self-oscillation vary with gravitational acceleration $\bar{g}$. As $\bar{g}$ increases, the A initially rises and then falls, while f has barely changed at all. It is noteworthy that $\bar{g}$ can be rewritten as $\bar{g}={\left(\tau /\tau_{0}\right)}^{2}$, where $\tau_{0}=\sqrt{L_{0}/g}$ represents the natural period of the LCE fiber-slider system. Thus, $\bar{g}$ reflects the relative speed of thermal relaxation compared to natural oscillation. For a small $\bar{g}$, thermal relaxation occurs too rapidly, causing the recovery of photo-induced contraction to happen almost exclusively at the moment the light source is turned off. Conversely, for a large $\bar{g}$, the thermal relaxation is too slow, resulting in a minimal recovery of photo-induced contraction when the light source is switched off. Consequently, when the light source is turned back on, the increase in photo-induced contraction is also insufficient. Therefore, whether $\bar{g}$ is too small or large, the net work done by the tensile force of the LCE fiber is inadequate to compensate for the energy dissipated, preventing sustained oscillation. These results suggest that a suitable increase in $\bar{g}$ within a specific range can enhance the oscillatory amplitude and improve the conversion efficiency of light energy into mechanical energy in engineering applications.

### 4.7. Impact of the Track Position

**Figure 10:**
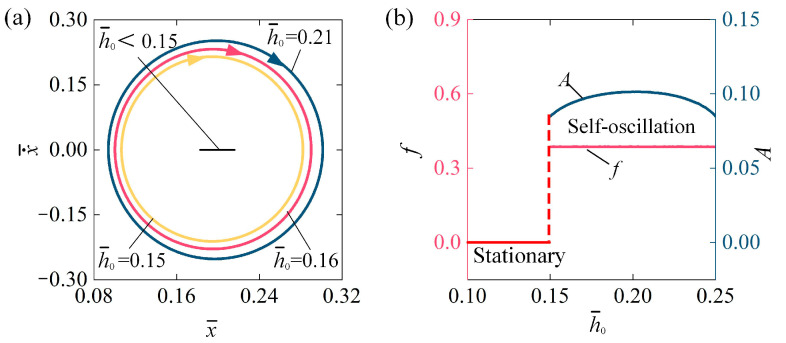
Influence of conductive track position on self-oscillation of LCE fiber-slide system, for ${\bar{I}}_{0}$ = 0.1, $C_{0}$ = 0.2, $\bar{\beta }$ = 0.1, ${\bar{v}}_{0}$ = 0.1, $\bar{k}$ = 5.8, and $\bar{g}$ = 1.2. (**a**) Stable cycles. (**b**) f and A. As ${\bar{h}}_{0}$ increases, the A initially rises and then falls, while f has barely changed at all.

Figure 10 describes the influence of conductive track position ${\bar{h}}_{0}$ on the self-oscillation of the LCE fiber-slide system, with parameters set to ${\bar{I}}_{0}$ = 0.1, $C_{0}$ = 0.2, $\bar{\beta }$ = 0.1, ${\bar{v}}_{0}$ = 0.1, $\bar{k}$ = 5.8, and $\bar{g}$ = 1.2. The stable cycles for various conductive track positions are shown in Figure 10a. The critical conductive track position ${\bar{h}}_{0}$ required to initiate the self-oscillation of the LCE fiber-slide system is approximately ${\bar{h}}_{0}$ = 0.15. In other terms, for ${\bar{h}}_{0}$ < 0.15, the system is in a stationary phase. For ${\bar{h}}_{0}$ = 0.15, ${\bar{h}}_{0}$ = 0.16, and ${\bar{h}}_{0}$ = 0.21, the system demonstrates a self-oscillation phase. Figure 10b illustrates how the f and A  of self-oscillation and conductive track position ${\bar{h}}_{0}$ change. As ${\bar{h}}_{0}$ increases, the A initially rises and then falls, while f has barely changed at all. The principle of influence of ${\bar{h}}_{0}$ can be summarized as follows: for a small ${\bar{h}}_{0}$, the slider easily activates the light source in the conductive segment, resulting in significant contraction of the LCE fiber. However, it must travel a considerable distance before the insulating segment makes contact with the track to turn off the light and allow the fiber to recover, which is limited by the weight of the slider. For a large ${\bar{h}}_{0}$, the slider needs to move down a significant distance to activate the light source adequately. In this case, the tension in the LCE fiber also restricts how far the slider can move downward. Therefore, whether ${\bar{h}}_{0}$ is too small or large, the energy taken in by the LCE fiber-slider system is insufficient to offset energy loss, preventing sustained oscillation. Thus, adjusting ${\bar{h}}_{0}$ within a certain range appropriately can enhance the A, thereby improving the effectiveness of converting optical energy for practical engineering purposes.

## 5. Conclusions

Self-oscillation efficiently transforms constant external inputs into mechanical energy, making it an excellent method for sustaining continuous motion. This characteristic forms the foundation for its broad application in engineering. Most self-oscillation systems have been developed by utilizing a static external environment that continuously releases energy. However, materials only intermittently absorb ambient energy, resulting in significant energy waste. To address this issue, we introduce a new self-oscillation of the LCE fiber-slide system driven by a self-flickering light source, which can regulate the energy source in accordance with the self-oscillating behavior. This system primarily consists of a photo-responsive LCE fiber, a slider that includes a conductive segment and an insulating segment, a light source, and a conductive track. By employing the dynamic LCE model, we derive the governing equation for the motion of the LCE fiber-slider system.

Through simulation, we identify two motion phases: the stationary phase and the self-oscillation phase. The self-oscillation of the LCE fiber-slider system arises from the photo-induced contraction of the LCE fiber when the light source is activated. We also investigate how the system parameters impact self-oscillation behavior, including critical conditions, frequency f, and amplitude A. The A primarily depends on parameters such as ${\bar{I}}_{0}$, $C_{0}$, $\bar{\beta }$, $\bar{k}$, $\bar{g}$, and ${\bar{h}}_{0}$. Increasing system parameters, including ${\bar{I}}_{0}$, $C_{0}$, and $\bar{k}$, can enhance both the f and A. As $\bar{g}$ increases, the A shows a pattern of gradually rising to a maximum point and then steadily decreasing, while the f  has barely changed at all. Moreover, the change in A with increasing ${\bar{h}}_{0}$ follows a pattern identical to that of $\bar{g}$; conversely, an increase in $\bar{\beta }$ results in a decrease in A of self-oscillation. The f is primarily determined by $\bar{k}$. There exists a crucial ${\bar{v}}_{0}$ that triggers the self-oscillation, but ${\bar{v}}_{0}$ has no significant impact on the A  and f.

Unlike conventional photo-induced self-oscillating systems with continuous illumination, our design features a dynamic self-flickering light source that interacts with the oscillation of the conductive slider under constant voltage, facilitating periodic switching over time. This configuration provides several benefits, including spontaneous periodic lighting, a streamlined structure, energy efficiency, and ease of use. It shows great potential for applications in dynamic circuit systems, monitoring and warning devices, optical devices, and more. It is important to acknowledge that the model proposed in this study has certain limitations, including the assumption of negligible friction between the slider and the track, a linear damping force, and a homogeneous tensile force proportional to the elastic strain, among others. In future work, we may investigate the effects of these factors or consider the use of more advanced models and illustrate the self-oscillation phenomenon through experimental validation to confirm the numerical calculations. We believe that this work has the potential to inspire new and diverse design concepts for dynamic circuit systems, monitoring and warning devices, optical devices, and more.

## Figures and Tables

**Table 1:** Material properties and geometric parameters.

Parameter	Definition	Value	Units
$C_{0}$	Contraction coefficient	0~0.5	/
$I_{0}$	Light intensity	0~0.15	$kW/m^{2}$
τ	*trans*-to-*cis* thermal relaxation time	1~100	ms
$\eta_{0}$	Light absorption constant	0.0003	$m^{2}/(s\cdot W)$
$L_{0}$	Original length of LCE fiber	1~10	cm
k	Stiffness coefficient	0~20	N/m
g	Gravitational acceleration	10	$m/s^{2}$
β	Damping coefficient	0~0.6	mg·mm/s
m	Mass of the slider	0~10	g
$h_{0}$	Position of the conductive track	0~0.1	m
$v_{0}$	Initial velocity	0~0.1	m/s

**Table 2:** Dimensionless parameters.

Parameter	${\bar{I}}_{0}$	$\bar{\beta }$	$\bar{k}$	${\bar{v}}_{0}$	$\bar{g}$	${\bar{h}}_{0}$
Value	0~0.5	0~0.5	0~10	0~0.2	0.1~2	0~0.3

## Data Availability

The original contributions presented in the study are included in the article, further inquiries can be directed to the corresponding author.
